# Peripheral Immune Cells Exhaustion and Functional Impairment in Patients With Chronic Hepatitis B

**DOI:** 10.3389/fmed.2021.759292

**Published:** 2021-10-26

**Authors:** Xin Jin, Zhi-han Yan, Lingna Lu, Shengjia Lu, Guoping Zhang, Wei Lin

**Affiliations:** ^1^Department of Clinical Laboratory, Tongde Hospital of Zhejiang Province, Hangzhou, China; ^2^Department of Hepatology, Wuxi Fifth People's Hospital, Wuxi, China; ^3^Department of Infectious Diseases, Tongde Hospital of Zhejiang Province, Hangzhou, China; ^4^Department of Clinical Laboratory, The Affiliated Suzhou Hospital of Nanjing Medical University, Suzhou Municipal Hospital, Gusu School, Nanjing Medical University, Suzhou, China; ^5^Department of Otolaryngology, Tongde Hospital of Zhejiang Province, Hangzhou, China

**Keywords:** chronic hepatitis B, immune cells, exhaustion, cytokines, functional impairment

## Abstract

After infection of hepatitis B virus (HBV), the virus induces a variety of immune disorders in the host, leading to immune escape and, finally, the chronicity of the disease. This study investigated immune cell defects and functional impairment in patients with chronic hepatitis B (CHB). We analyzed the percentage, function, and phenotypes of various immune cell subpopulations in the peripheral blood along with the concentrations of cytokines in the plasma. We compared the results between patients with CHB and healthy individuals. It was found that in patients with CHB, the cell function was impaired and, there was increased expression of inhibitory receptors, such as NKG2A and PD-1 in both NK and T cells. The impairment of function was mainly in cytokine secretion, and the cytotoxicity was not significantly diminished. We also found that the proportion of dendritic cells (DC) decreased and regulatory B cells (Breg) increased in CHB. In addition, the Breg cells were negatively correlated with T cell cytokine and positively correlated with ALT and HBV viral load. Taken together, various disorders and functional impairments were found in the immune cells of peripheral blood in CHB patients, especially NK and T cells. These cells showed exhaustion and the increase of regulatory B cells may be one of the reasons for this phenomenon.

## Introduction

Despite the availability of highly effective preventive vaccines and oral antiviral drugs, chronic infection with hepatitis B virus (HBV) still affects more than 240 million people worldwide and causes 620,000 deaths annually (1, 2). HBV is a non-cytopathic hepatophilic double-stranded DNA virus (3). Most people develop an acute self-limiting disease with host immunity after infection with the virus. However, the virus can use various strategies to evade host surveillance (4, 5), and those patients who fail to clear the virus to develop chronic infection.

In patients with chronic hepatitis B (CHB), the host immune response is like a double-edged sword. On one hand, it achieves clearance of the hepatitis B virus by destroying infected hepatocytes, and on the other hand, the immune response causes liver inflammation and aggravates liver damage, leading to liver fibrosis and hepatocellular carcinoma (6, 7). In addition, the HBV virus can induce host immune dysfunction, causing immune imbalance and functional defects. The exhausted immune cells could accelerate infected hepatocytes to achieve immune escape and promote disease development because of their inability to perform immune monitor (8–11).

The immune system is extremely complex, and there is still no clear explanation on the antiviral inflammation of the body after HBV infection and on the immune escape of the virus. This study intends to investigate the possible dysfunction of the immune system in patients with chronic hepatitis B through the analysis of the percentage, phenotype, and function of peripheral immune cells in 48 patients with chronic HBV infection. Our purpose is to provide clinical data for a more in-depth understanding of the mechanisms of immunodeficiency associated with chronic hepatitis B infection and, furthermore, to offer a theoretical basis for immunotherapy in hepatitis B patients.

## Materials and Methods

### Patients and Controls

A total of 48 patients, which included 30 male and 18 female patients, with chronic hepatitis B (CHB) were recruited from the Department of Hepatology in Tongde Hospital of Zhejiang Province. The age of patients ranged from 16 to 72 years old with the mean age of 39 ± 14 years. These patients were HBsAg positive for longer than 6 months, volunteered to participate in the study, and had complete case information. None of the CHB patients had a history of liver surgery, coinfection with another viral hepatitis, autoimmune hepatitis, or human immunodeficiency virus (HIV) infection, and there were also no pregnant or lactating women. The patients were divided into immune tolerant (IT) phase (*n* = 13), immune clearance (IC) phase (*n* = 12), lower replicative (LR) phase (*n* = 13), and reactivation (RA) phase (*n* = 10) according to the Asian Pacific Association for the Study of Liver guidelines (12) (Table 1). In addition, 15 healthy control specimens from the health checkups, all of whom had excluded HBV infection, HCV infected or HIV infected, and had normal serum alanine aminotransferase (ALT) levels, were included in the study. CHB and healthy control characteristics are detailed in Table 2. All specimens were enrolled after obtaining informed consent from the patients or their families. The study was approved by the Ethics Committees of the Tongde Hospital of Zhejiang Province [identification nos. HMU (Ethics) 2017-K044].

**Table 1:** Clinical characteristics of 4 subgroups of patients with CHB.

**Characteristics**	**IT**	**IC**	**LR**	**RA**
Number	13	12	13	10
Age (years)	36 (25–68)	35 (17–72)	43 (28–59)	45 (16–72)
Gender (M/F)	7/6	8/4	8/5	7/3
ALT (IU/L)	26.8 (11–39)	174.6 (73–537)	24.8 (10–35)	233.3 (67–575)
HBV DNA log10 IU/mL	7.8 (7.4–8.2)	5.7 (3.8–8.6)	2.5 (2.1–3.3)	4.4 (3.5–7.1)
HBeAg positive/negative	13/0	12/0	0/13	0/10
ALT raised/normal	0/13	12/0	0/13	10/0

*IT, immune tolerant; IC, immune clearance; LR, lower replicative; RA, reactivation*.

**Table 2:** Clinical characteristics of CHB patients and HC.

**Characteristics**	**CHB**	**HC**
Number	48	15
Age (years)	36 (16–72)	38 (23–48)
Gender (M/F)	30/18	9/6
ALT (IU/L)	106.2 (10–575)	21.3 (11–35)
HBV DNA log10 IU/mL	5.1 (2.1–8.6)	NA
HBeAg positive/negative	25/23	NA
ALT raised/normal	22/26	0/15

*CHB, chronic hepatitis B; HC, healthy control; M/F, male/female; ALT, alanine transaminase; HBeAg, hepatitis B e antigen; HBV, hepatitis B virus; NA, not applicable*.

### Specimen Processing

The 2 ml peripheral blood was collected using the EDTA anticoagulated or heparin anticoagulated (for intracellular cytokine assay) tubes. The peripheral blood mononuclear cells (PBMC) were obtained by density gradient centrifugation by Ficoll separation. In addition, 5 ml of procoagulated blood was centrifuged at 1,000g for 10 min, the plasma was obtained and stored at −80°C for testing. All treatments were completed within 24 h.

### Analysis of Cell Surface Molecule Expression by Flow Cytometry

All analysis was performed in four protocols. Antigen-presenting cell protocol, CD16-FITC, CD11c-PE, CD56-PC5, CD123-PC7, CD14-APC, CD3-APC750, HLA-DR-PB, CD45-KO, B cell protocol, CD38-FITC, IGD-PE, CD19-PC5, CD CD27-PC7, CD24-APC, T cell protocol, CD45RA-FITC, CD127-PE, CD25-ECD, PD-1-PC5, CD45-PC7, CD4-APC, CD8-APC700, CD3-APC750, CXCR5-PB. NK cell, CD16-FITC, CD8-PE, CD56-PC5, CD45-PC7, NKG2A-APC, CD3-APC750, and NKp30-PB. All antibodies were added with 5 ul. Then each protocol was added 50 ul PBMC, incubated for 20 min in the dark, added 500 ul hemolysin, hemolyzed for 5 min, centrifuged, and discarded supernatant before resuspended. Flow cytometry assay and set up isotype control were performed. All assays were completed within 1 h after isolation of PBMC.

### Test of Granzyme and Perforin

Firstly, the cell surface staining was performed by taking 50 ul of PBMC, adding 5 ul to each of CD56-PC5, CD45-PC7, CD4-APC, CD8-APC-700, and CD3-APC-750 antibodies, respectively, and incubating for 20min in the dark. Then, hemolysin, fixative, and membrane breaker were added in order, with 20 min of incubation and centrifugation after the addition of each agent. Finally, 5 ul of perforin -FITC and granzyme-PE antibody was added into each mixture, vortexed, and incubated for 20 min in the dark.

### Intracellular Cytokine Staining

We analyzed intracellular cytokines in 31 of the 48 CHB patients. Cells were stimulated for 3 hours in disposable dry powder tubes precoated with stimulating agents (PMA, ionomycin, brefeldin) at 37°C in 5% CO_2_, then stained with surface markers CD45-PC7, CD3-APC-750, CD4-APC, CD8-APC700, and CD56-PC5 while being protected from light for 15 minutes. These were then fixed and broken to stain intracellular cytokines IFN-γ-FITC, IL-21-PE, and IL-17-PB, before finally being detected by flow cytometry.

### Multiplex Cytokine Cytometric Bead Array

Plasma concentration of cytokines was tested in the 31 patients with CHB. Plasma (standard), buffer, mixed capture beads, and detection antibody, each with affinity 25 ul was mixed and incubated for 2 h in the dark. Then, streptavidin with fluorescein was added (SA-PE) and mixed in the dark for half an hour before being centrifuged. The supernatant was then discarded before 100 ul of PBS was added. Finally, the flow cytometer was tested. The results were quantified in the software LEGEND plex V8.0 for analysis.

### Instrument and Reagents

Antibodies: CD279 (PD1) (Clone PD1.3 Beckman Coulter), CD45RA (Clone ALB11 Beckman Coulter), CD185 (CXCR5) (Clone J252D4 Biolegend), CD4 (Clone 13B8.2 Beckman Coulter), CD20 (Clone B9E9 Beckman Coulter), CD19 (Clone J4.119 Beckman Coulter), CD38 (Clone HB-7 Biolegend), CD27 (Clone M-T271 Biolegend), IGD (Clone IA6-2 Biolegend), CD11c (Clone BU15 Beckman Coulter), CD14 (Clone RMO52 Beckman Coulter), CD16 (Clone 3G8 Beckman Coulter), CD24 (Clone ALB9 Beckman Coulter), CD45 (Clone J.33 Beckman Coulter), CD3 (Clone UCHT1 Beckman Coulter), CD8 (Clone B9.11 Beckman Coulter), CD56 (Clone N901 Beckman Coulter), CD57 (Clone NC1 Beckman Coulter), CD25 (Clone B1.49.9 Beckman Coulter), CD127 (Clone R34.34 Beckman Coulter), CD159a (NKG2A)(Clone S19004C Biolegend), CD337 (NKp30) (Clone P30-15 Biolegend), IFN-γ (Clone 45.15 Beckman Coulter), IL-21 (Clone 3A3-N2 Biolegend), IL-17 (Clone BL168 Beckman Coulter), granzyme (Clone QA16A02 Biolegend), perforin (Clone B-D48 Biolegend)

Flow Cytometer: Navios, Beckman Coulter

Cytometric Bead Array: RAISECARE; Analysis Software: Kaluza, LEGEND plex.

### Laboratory Indices

Alanine aminotransferase activity in serum was measured by an auto biochemical analyzer (AU5800, Beckman Coulter), with the reference range of 9–50 IU/L. The HBV markers, HBsAg and HBeAg, were determined by commercially available enzyme-linked immunosorbent assays (Shanghai KH Biology). Serum HBV DNA was quantitated by real-time quantitative polymerase chain reaction using a commercially available kit (Anadas9850, Amplly), the lower detection limit was 30 IU/ml.

### Statistical Analysis

Statistical analysis was performed using SPSS 22.0 Software (IBM, USA). Data were expressed as the mean ± standard deviation and *n* (%). Student's *t*-test or One-way ANOVA was conducted to compare two groups and comparisons between three or more means. Correlations between variables were calculated with the Spearman rank correlation test. In our studies, *p* < 0.05 were considered significant.

## Results

### The Proportion or Function of Antigen-Presenting Cells Decreased in Patients With CHB

**Figure 1:**
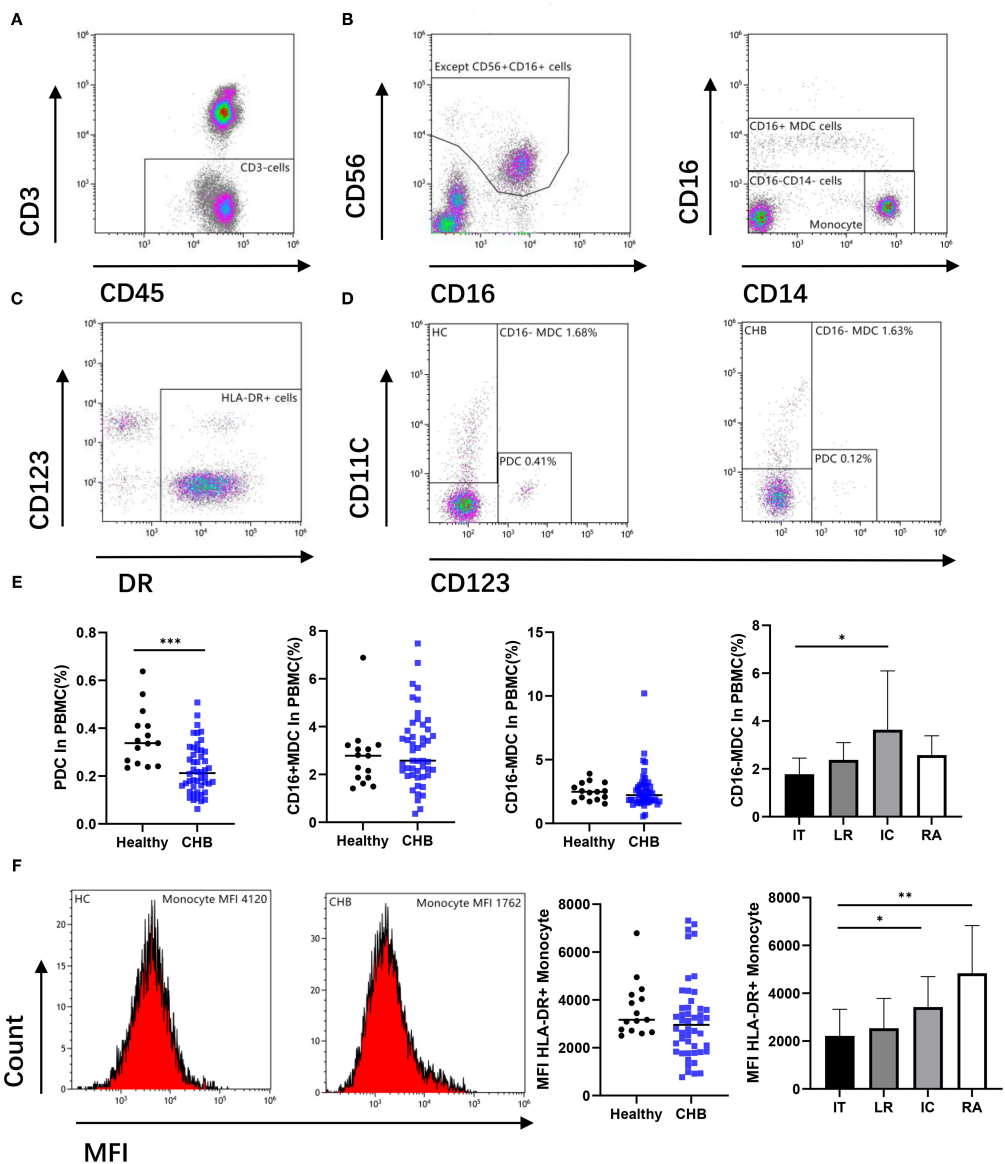
Antigen-presenting cells (APCs) were altered during chronic hepatitis B virus (HBV) infection. **(A,B)** Remove the CD56 positive cell from the CD3 negative gate, and the remaining parts were divided into CD16+ myeloid dendritic cell (MDC), Monocyte, and CD16–CD14– cell. **(C,D)** Circle the DR positive cells from CD16 and CD14 double negative cells, there are CD123+ (plasma dendritic cell, PDC) and CD11c+ (CD16– MDC) cells among them. Representative flow cytometry plot indicates the percent frequencies of PDC as well as CD16– MDC in healthy (*n* = 15) and CHB patients (*n* = 48) respectively. **(E)** According to different alanine aminotransferase (ALT) and HBV DNA levels, all chronic hepatitis B (CHB) were divided into immune tolerant (IT; *n* = 13), lower replicative (LR; *n* = 13), immune clearance (IC; *n* = 12) and reactivation (RA; *n* = 10). The percentages of PDC and MDC in different groups were compared. **(F)** Mean fluorescence intensity (MFI) of DR in CD14+CD16– Monocyte cell was compared. Each data point represents an individual subject. Horizontal lines show the median. Statistically significant differences are indicated by ^*^p < 0.05; ^**^p < 0.01; ^***^p < 0.001.

To determine whether deficiency of antigen-presenting cells (APC) occurs in patients, we investigated the percentage of dendritic cells (DC) in patients with CHB and compared it with the healthy controls. The identification of DC by flow cytometry was shown in Figure 1. Our result indicates that the plasma dendritic cell (PDC) in patients with CHB was lower than those in the healthy group (*p* < 0.001). However, CD16+ and CD16– myeloid dendritic cells (MDC) do not show a significant difference between these two groups. In order to explore whether there are changes between different stages of CHB, we further compared the DC in CHB with different stages, and we found that CD16– MDC in the IT group was lower than that in IC (*p* = 0.026) group (Figure 1E), PDC and CD16+ MDC have no difference among each stage (data not shown).

As another important role of APC, we also compared the mean fluorescence intensity (MFI) of HLA-DR expression in monocyte cells. There was no difference found between CHB and the normal group. However, in different stages of CHB, results show significantly lower MFI expression in IT than that in IC (*p* = 0.019) and RA (*p* = 0.002) stage (Figure 1F).

### Peripheral Blood Breg Elevated in CHB Patient

**Figure 2:**
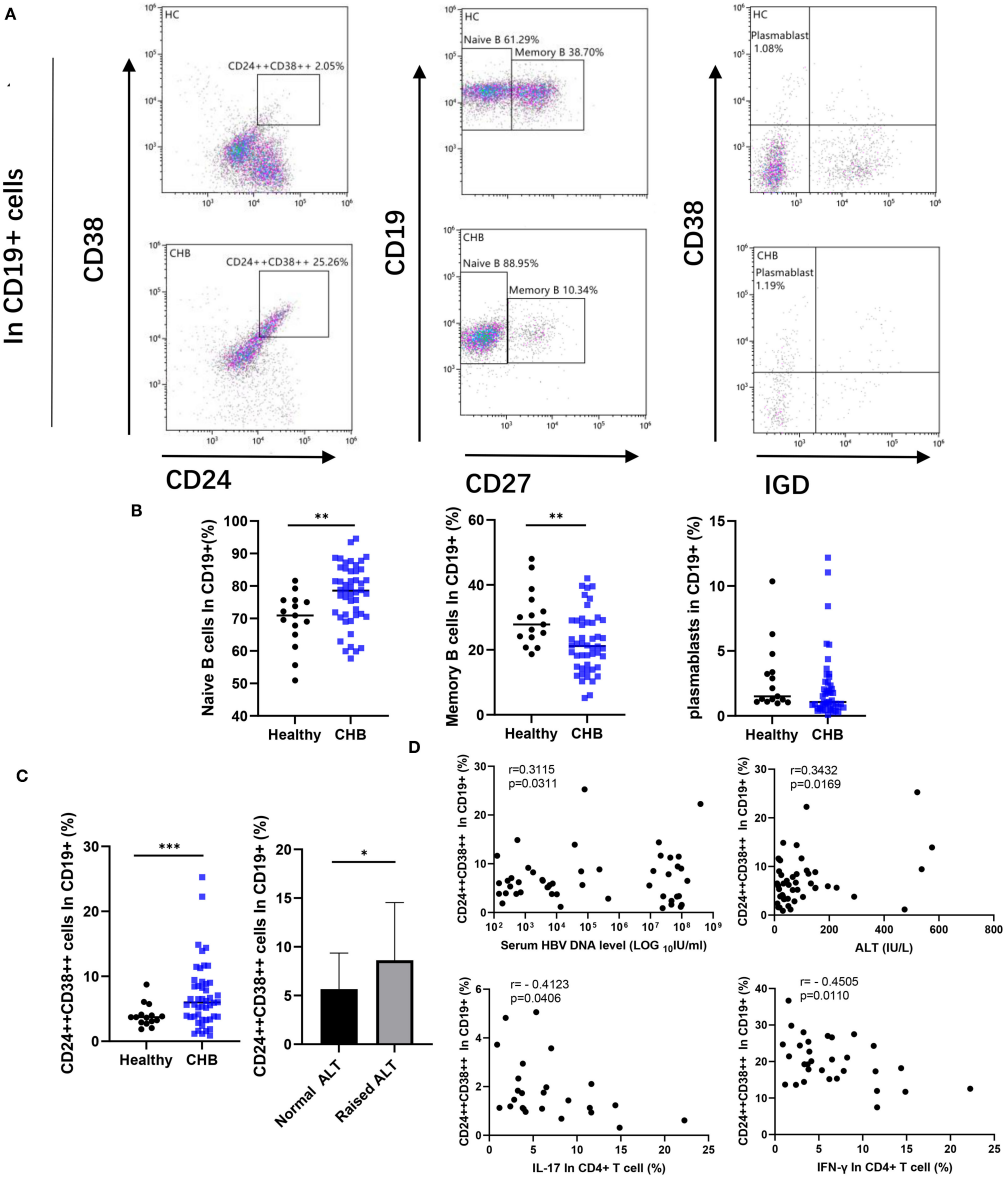
Comparative analysis of peripheral B cell subsets. **(A)** A representative dot plot of B cell subsets in healthy and chronic hepatitis B (CHB). The cells strongly expressed CD24 and CD38 were defined as Regulatory B cells (Breg). We circled CD19+CD27– subset as Naïve B cell, and CD19+CD27+ cells as Memory B cell. The CD19+CD27+ cells further divided into Plasmablast (CD38+IgD–) according to the expression of surface marker CD38 and IgD. **(B)** Percentage of each subset among CD19+ B cells in healthy controls (*n* = 15), and CHB (*n* = 48) were compared. **(C)** Percentage of Breg in CHB was compared with the healthy controls, meanwhile the difference between normal (*n* = 26) and abnormal alanine aminotransferase (ALT; *n* = 22) in CHB patients was also showed. **(D)** The correlation among the Breg, the concentration of ALT, HBV DNA in serum of CHB patient (*n* = 48), and cytokines secreted by CD4+ T cells (*n* = 31) as shown in the figure. Statistically significant differences are indicated by ^*^p < 0.05; ^**^p < 0.01; ^***^p < 0.001.

We compared B cell subsets in the peripheral blood of healthy control and CHB patients. We circled the plots of highly expressed CD38 and CD24 in CD19+ cells, which we considered as regulatory B cells (Breg) (Figure 2A). Our data revealed that the number of Breg in CHB was higher than that in the healthy group (*p* < 0.001). The CHB patients with abnormal ALT were higher than normal ones (*p* = 0.040) (Figure 2C). In addition, Breg cells were positively correlated with the concentration of ALT and HBV DNA in CHB (*p* = 0.017 and *p* = 0.031, respectively), and negatively correlated with IL-17 and IFN-γ that was secreted by CD4+ T cells (*p* = 0.041 and *p* = 0.011, respectively) (Figure 2D). In comparison to other subclasses, we found that the proportion of CD27– Naïve B cells decreased in CHB (*p* = 0.005), on the contrary, the Memory B cells increased (*p* = 0.005). There was no significant difference in plasma blast between healthy and CHB groups (Figure 2B).

### Circulating T Follicular Helper Cell Increased in CHB, and Treg in Peripheral Blood Did Not Have a Difference in Healthy Control

**Figure 3:**
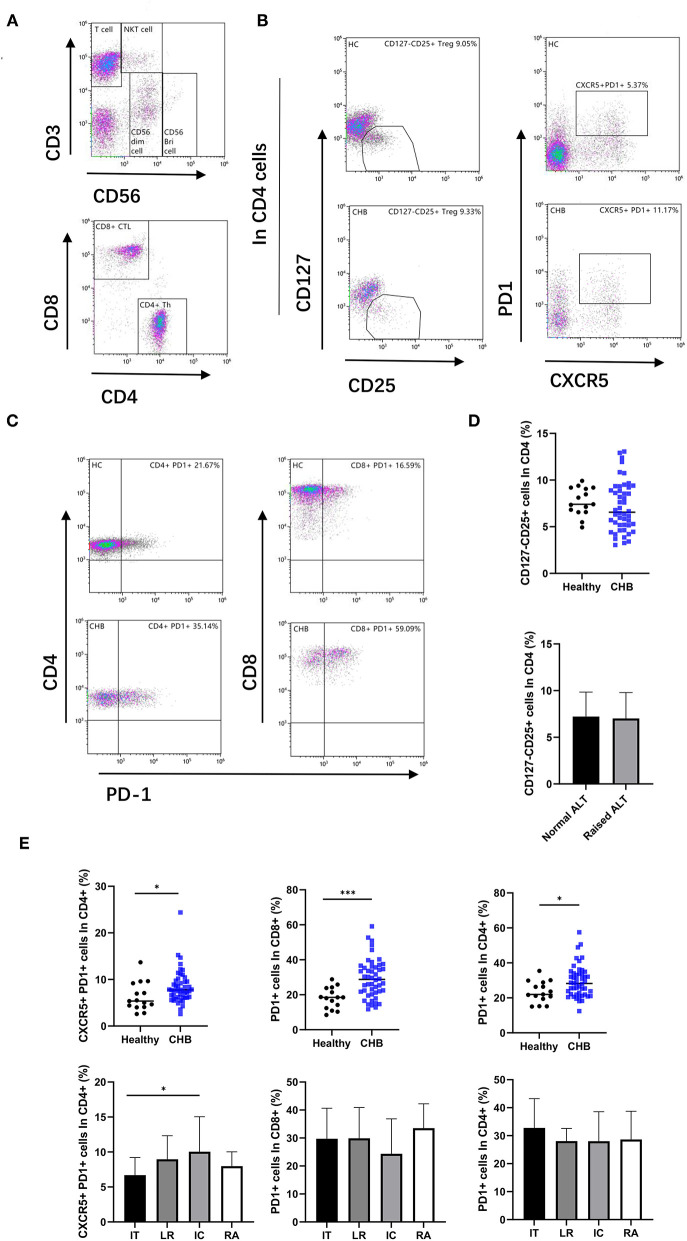
Alter T cell subsets in CHB patient. **(A)** According to the expression of CD3 and CD56, lymphocytes were divided into T cell, CD56 bright NK cell, CD56 dim NK cell, and NKT cell, and then, the T cells were divided into CD4+ Th cell and CD8+ CTL cell. **(B)** The representative flowcytometry plot indicates the percent frequencies of Treg and cTFH cells in healthy controls and CHB patients, respectively. The gating strategy for the analysis of Treg is CD4+CD127–CD25+, and CD4+PD1+CXCR5+ plots defined as the cTFH. **(C)** Representative plot of PD-1 expression on CD4 and CD8 T cells in different groups. **(D)** Comparison frequencies of Treg between healthy controls (*n* = 15) and CHB patients (*n* = 48), and that in ALT normal (*n* = 26) and abnormal patients (*n* = 22). **(E)** The percentage of cTFH and PD-1 positive cells in different groups were shown. Immune tolerant (IT; *n* = 13), lower replicative (LR; *n* = 13), immune clearance (IC; *n* = 12) and reactivation (RA; *n* = 10). Statistically significant differences are indicated by ^*^p < 0.05; ^***^p < 0.001.

The CD4+Th subsets were further analyzed. The CD127–CD25+ cells were defined as regulatory T cells (Treg). The cells with high expression of CXCR5 and PD-1 were regarded as circulating T follicular helper cells (cTfh) (Figures 3A,B). Our results revealed that there was no significant difference of Treg in peripheral blood between CHB and healthy controls or between CHB patients with abnormal and normal ALT (Figure 3D). In the analysis of cTfh, we found that the ratio in CHB was higher than in healthy patients (*p* = 0.035). Similarly, in a different stage of CHB, cTfh in IC was higher than in IT patients (*p* = 0.043) (Figure 3E).

### Increased PD-1 Expression in T Cells

We examined the expression of programmed cell death protein 1 (PD-1) in T cells (Figure 3C). The results indicate that PD-1 expression on both CD4+ and CD8+ T cells was significantly higher in CHB patients than in healthy controls (*p* = 0.019 and *p* < 0.001, respectively), suggesting increased T cell exhaustion in CHB patients. Interestingly, we did not find differences in PD-1 expression in the comparison of different stages of CHB (Figure 3E).

### NK Cells Functionality Impaired in CHB and Displayed an Altered Phenotype

**Figure 4:**
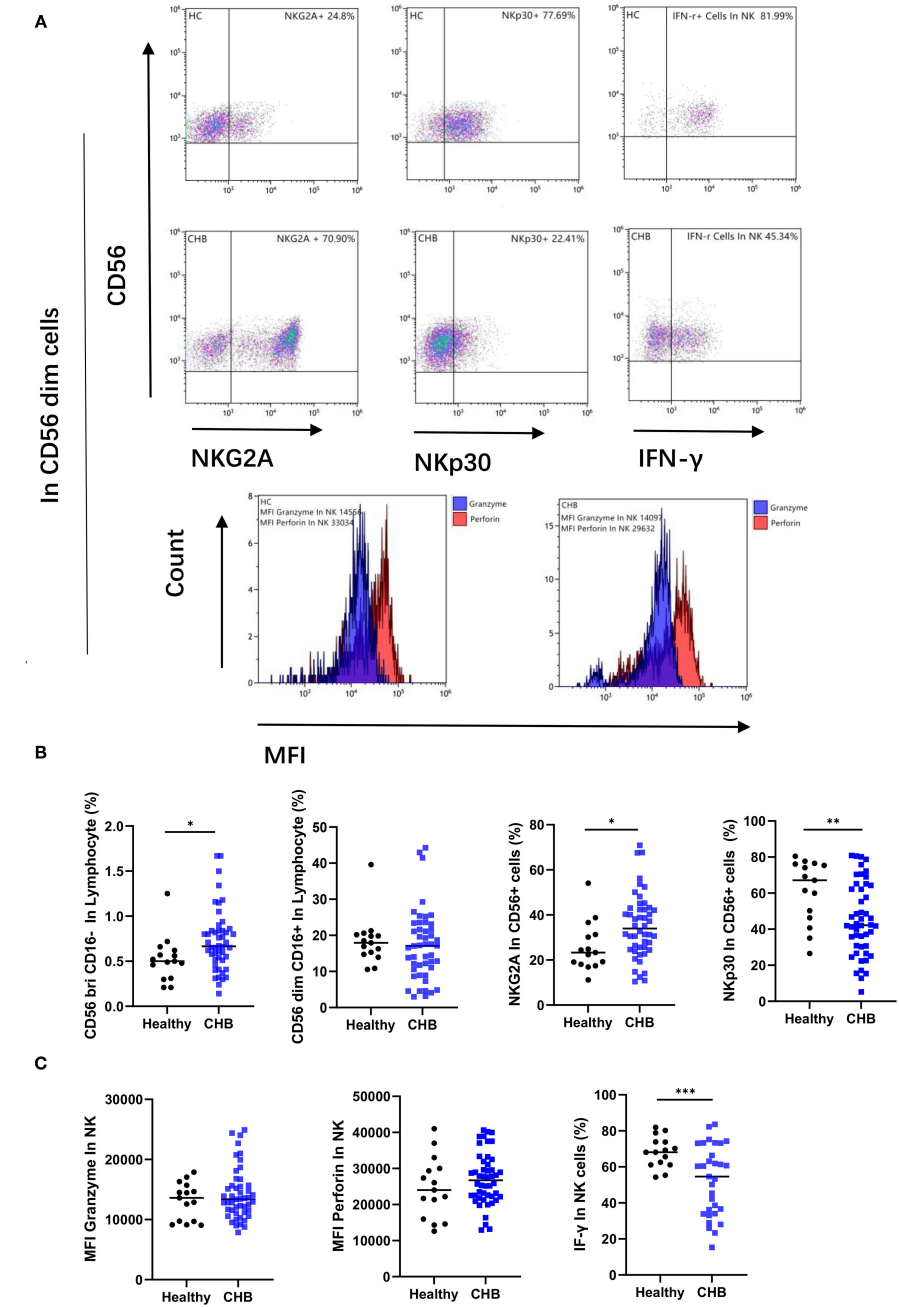
Study of the phenotype and function of natural killer (NK) cells in chronic hepatitis B (CHB) patients. **(A)** Typical results showing, the expression of the NK receptor, and the secretion of cytokine by NK cells, as well as the Mean fluorescence intensity (MFI) of the granzyme (blue) and perforin (red) in NK cells. **(B)** Comparison of NK cell numbers and phenotypes in CHB patients (*n* = 48) and healthy controls (*n* = 15), including the numbers of two subpopulations with high and low CD56 expression, and the expression of NK inhibitory receptor NKG2A and activating receptor NKp30. **(C)** Comparison of NK cell granzyme, perforin (*n* = 48), and IFN-γ secretion functions in CHB (*n* = 31) and healthy control groups (*n* = 15).

We measured the percentage of subsets of immune cells defined by CD56 expression levels. We observed that there was an increase in the proportion of NK cells in the group of CD56 positive in CHB patients (*p* = 0.038), while the number of CD56 dim subsets did not change (Figure 4B). The CD56 dim NK cell effector capacities were then analyzed with two different functions: cytotoxicity and the production of cytokines. Our results showed that the MFI of granzyme and perforin, which represent the cytotoxicity function of NK cells, was not significantly different in CHB and healthy controls. But the function of IFN-γ secretion by NK cells was decreased in CHB (*p* < 0.001) (Figures 4A,C). In order to determine whether the poor functionality could be explained by the altered expression of certain receptors, we characterized the phenotype of NK cells in CHB patients compared to healthy controls. The results of the study revealed that the inhibitory receptor NKG2A is elevated (*p* = 0.025) and, in contrast, the activating receptor NKP30 is decreased (*p* = 0.009) in CHB patients (Figures 4A,B).

### Enhanced T-Cell Cytotoxicity and Impaired Cytokine Secretion in CHB Patients

**Figure 5:**
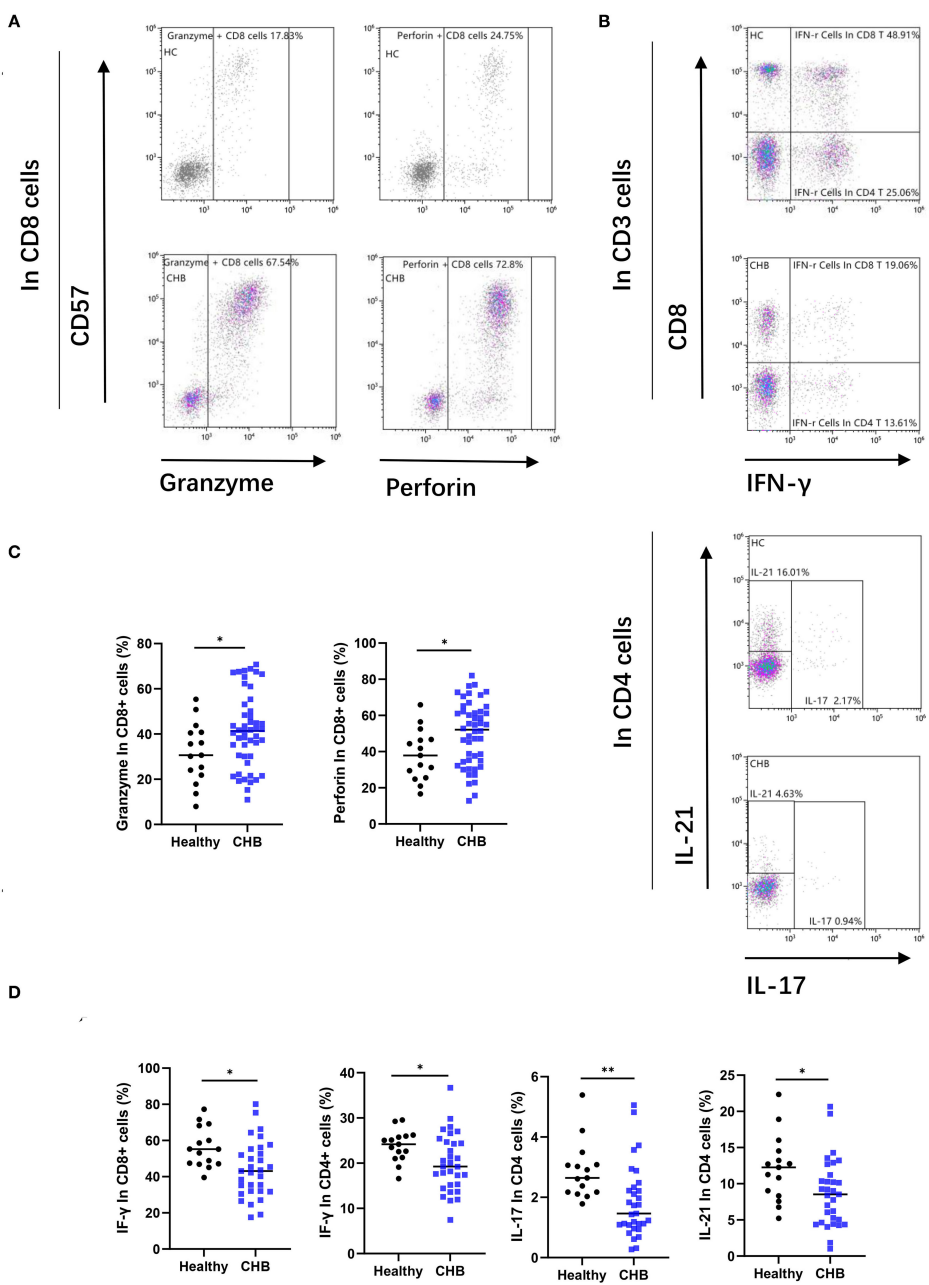
Chronic hepatitis B (CHB) patients show functional alteration in T cells. **(A)** Representative flow-cytometry plots, as well as the proportion of granzyme and perforin in CD8+T cells, are shown. **(B)** Peripheral blood mononuclear cells (PBMCs) from healthy controls and CHB patients were incubated for 3 h after stimulating by specialized tubes containing stimulating agents (PMA) and ionomycin. The proportion of CD8+ CTL cells positive for IFN-r and the gating strategy for analyzing the cytokine secretion capacity of CD4+ TH cells are shown in representative flow-cytometry plots. **(C)** Comparison of granzyme and perforin expression in CD8+ cells in healthy controls (*n* = 15) and CHB patients (*n* = 48). **(D)** Comparison of the functions of various types of cytokine secretion by T cells subset between healthy controls (*n* = 15) and CHB patients (*n* = 31).

To investigate the alterations of T cells functions in CHB disease. We also analyzed the two main functions, cytotoxic and cytokine secretion of T cells. As shown in Figures 5A–C, T cells from CHB patients presented higher expression of granzyme and perforin than controls (*p* = 0.038 and *p* = 0.024, respectively). This result suggested enhanced cytotoxic function of T cells in CHB. In contrast, cytokine secretion was impaired in CHB patients, and our results showed that the IFN-γ positivity in CD8+ T cells and the proportion of IFN-γ, IL-21, IL-17 in CD4+ T cells were all significantly lower than those in healthy controls (*p* = 0.038, *p* = 0.014, *p* = 0.024, and *p* = 0.006, respectively) (Figure 5D).

### IL-2 and IL-6 in Plasma Increased in CHB

**Figure 6:**
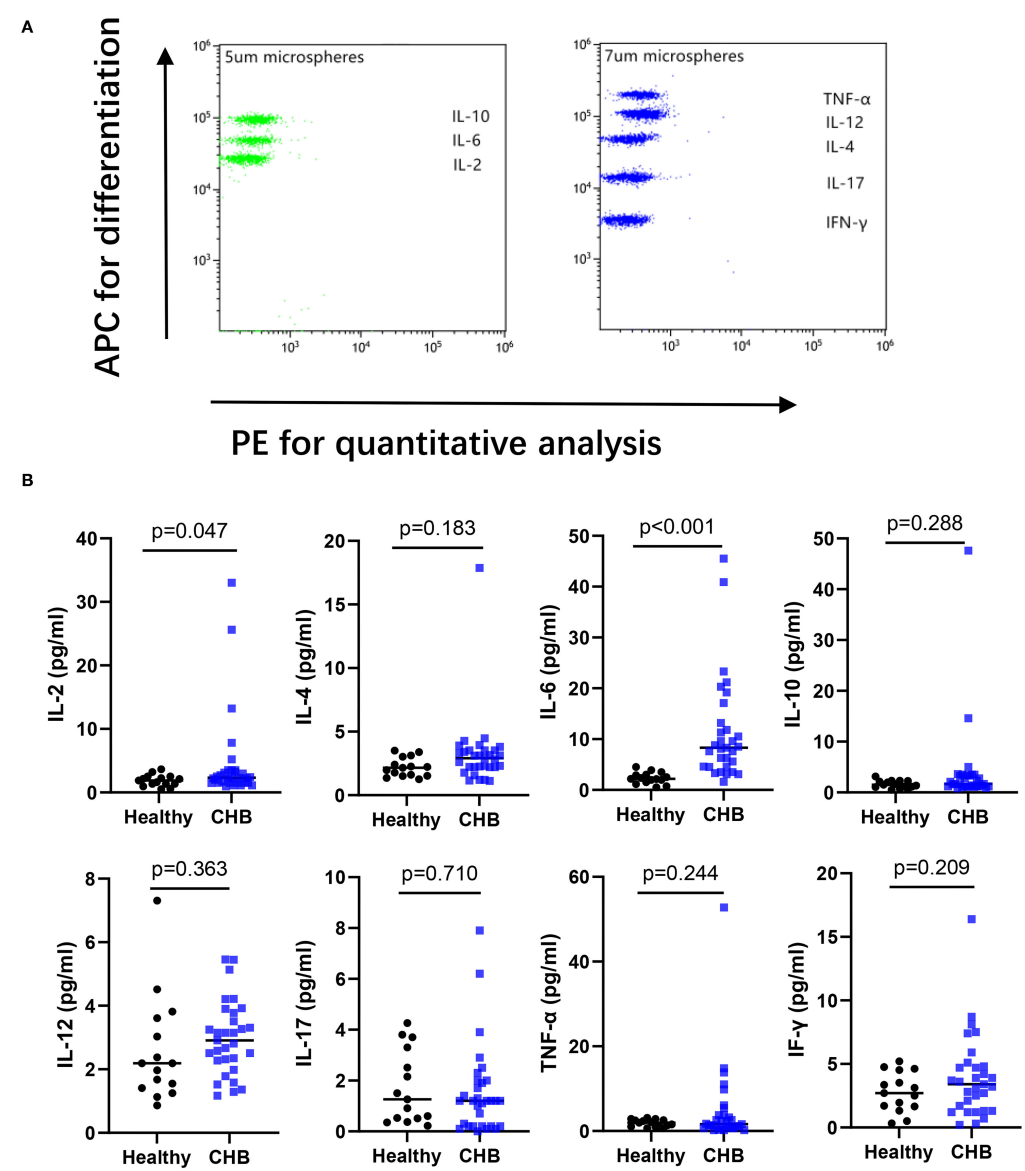
Analyzing the cytokines in plasma by flow cytometry. **(A)** An example of cytokine test by Cytometric Bead Array from healthy controls. **(B)** The concentration of IL-17, IFN-γ, IL-2, IL-6, TNF-α, IL-12, IL-10, IL-4 between healthy (*n* = 15), and CHB patients (*n* = 31) were compared.

The concentrations of cytokine in plasma were measured by Cytometric bead array (CBA). The capture antibodies for IL-2, IL-6, and IL-10 were coated on 5 um microspheres differentiated by three different levels of APC fluorescence intensity, respectively. IFN-γ, IL-17, IL-4, IL-12, and TNF-α were coated on 7 um microspheres of five APC fluorescence intensity levels in turn, and the PE channel was used for color development before being converted to cytokine concentration on the standard curve (Figure 6A). We found increased concentrations of IL-2 and IL-6 in the plasma of CHB patients, that were significantly different in the comparison of data from healthy controls (Figure 6B). We further compared the IL-2 and IL-6 in different immune phases, and the results showed that IL-2 in the IT group was lower than that in IC (*p* = 0.043), no significant differences were found in IL-6 across the four immune phases (data not shown).

## Discussion

Natural Killer cells account for ~5–15% of peripheral blood lymphocytes (13). It is the main immune cell of the body to deal with a viral infection. They can directly attack the infected cells through the cytotoxic functions of intercellular contents, and they can secrete multiple cytokines, such as interferon-γ (IFN-γ). CD3–CD56 + NK cells identified by flow cytometry can be further subdivided into NK cells of CD56dim and CD56 positive (14). The former is the main group that makes up NK cells, expressing CD16 (Fc receptor) and KIR receptors, playing a cytotoxic role, and the latter main function is the secretion of cytokines (15). Although CD56brightNK cells account for very little in the blood, this distribution can be significantly altered by persistent viral infection (16). Our study showed increased CD56brightNK cells in CHB patients, while CD56dimNK cells did not differ from normal, which was consistent with some of the previous literature reports (17).

Natural Killer cells are activated during acute HBV infection before the onset of adaptive immunity, and the effective immune response of NK cells leads to initial control of the acute infection in the early stages (18). However, when the disease enters a chronic stage, NK cells do not necessarily undergo physical loss but remain in a state of “exhaustion” with poor or no function (19). In the peripheral blood NK cell function test in the disease group, we found that the cytotoxicity of NK cells and the mean fluorescence intensity of protein in CHB patients were not significantly different from those in the healthy controls, while the function of IFN-γ secretion was significantly decreased, indicating impaired NK cell function. The phenomenon that the cytotoxic effect remains unchanged or even increases while the cytokine production ability decreases is called the “functional dichotomy” (16) by some scholars, and this functional defect may be more conducive to the survival of the virus. It is unfortunate that in the study of the cytokine secretion function, we only performed experiments on 31 specimens and did not discuss more detail in different immune phases of CHB, which is a limitation of this study, and we will do further analysis in subsequent research.

The function of NK cells is closely regulated by activating and inhibiting receptors. The interactions between NK cell receptors and corresponding ligands determine the state of NK cells (20). In a chronic viral infection, the function of NK cells may be impaired by changes in their receptors (21). To determine whether poor NK cell function in CHB patients can be explained by altered expression of activated or inhibitory receptors, we examined NK cell membrane surface receptors. We found that the expression of the NK cell-activating receptor NKp30 was significantly decreased compared with normal controls, while the expression of the inhibitory receptor NKG2A was increased in CHB patients.

Interestingly, it was found that T cells also had “exhaustion” in CHB patients. On one hand, the inhibitory receptor PD-1 of both CD4+ and CD8+ T cells was significantly increased, and on the other hand, the ability of each T cell subpopulation to secrete cytokines was decreased to varying degrees. More importantly, we also found a phenomenon similar to NK cells “functional dichotomy” in T cells, and our results showed the higher cytotoxic function of CTL than healthy controls. Studies have shown that dysfunctional NK CHB cells may exhibit the same molecular characteristics at both transcriptional and protein levels, and it is a kind of signal involved in calcium balance characteristics (22), which may also explain the consistency of “exhaustion” of NK cells and T cells in our results.

Immunoinhibitory cytokines and regulatory cells are also involved in the lymphocyte “exhaustion” process of CHB, and negative regulation is an important factor in the induction of CD8+ T and NK cell exhaustion (23–25). For example, it has been found that NKG2A + NK cell dysfunction comes from Tregs derived IL-10. Blockade of IL-10 leads to the reduction of NKG2A + NK cells and increases IFN-γ + NK cells (26). Our study defines the CD4 + CD25 + CD127-phenotype as Treg cells while CD19 + CD24+ + CD38+ + is the Breg cells. After comparing with the two major regulatory cells of peripheral blood, we found no significant changes in Treg in patients with CHB. There is a consistent view in these articles that Treg cells in the liver of CHB patients are significantly increased and are positively correlated with the poor prognosis of the disease, while there are different reports on the change of the number of Treg in the peripheral blood (27–29). At the same time, Treg cells belong to a category with high heterogeneity. Current studies suggest that there are also subsets of immune cells that promote inflammation in Treg cells. Hence, simple CD4+CD25+CD127– cannot well identify the truly negatively regulated cell populations in Treg. Therefore, the role of Treg in CHB still needs further exploration (30).

On the other hand, in Breg analysis, we found that Breg was significantly increased in patients with CHB, which was positively correlated with ALT and DNA levels, and negatively correlated with cytokine secretion of T cells. In previous studies on CHB, studies on B cells mainly focused on antibody production, while other functions such as antigen presentation and immune regulation, which are closely related to immune tolerance and liver injury, were ignored (31). We speculated that long-term high viral load leads to an inflammatory response in the body, and in order to avoid the damage of inflammatory response to organs, the production of Breg cells is increased. The Breg can inhibit the inflammatory response and induce immune cell exhaustion, resulting in immune escape of the virus. It has been reported that Breg can inhibit IFN-γ production through IL-35 secretion (32), which is consistent with the view of this study.

We also compared the concentrations of plasma cytokines in CHB patients. Among the detected cytokines, the levels of IL-2 and IL-6 in CHB patients were increased, while the widely reported inhibitory factor, IL-10, was not significantly changed (19, 33), which may be limited by methodology. Different from ELISA used in some previous studies, this experiment adopted the liquid flow CBA method, which showed good linearity in the detection of high values of cytokines, while for low values, the fitting curve may not well reflect the true results. At the same time, there were also several cases of significantly increased IL-10 in the disease group, but the data distribution was skewed, and no statistically significant difference was found. As a common pro-inflammatory factor, the increased level of IL-6 has been reported in various infections and tumors, and there have also been studies on the antiviral mechanism of IL-6 in hepatitis B (34, 35). In general, IL-6 has high sensitivity and poor specificity. IL-2 plays an important role in the cytotoxic role of CD8+ T cells, which can bind to IL-2R to directly kill the infected liver cells (36). Our results showed an increase in IL-2 in CHB patients, particularly in the IC phase, suggesting that the pathway of T-cells killing viruses through IL-2 may exist in patients with chronic infection.

We found no difference in levels of IFN-γ in serum between healthy and CHB, which does not contradict previous studies showing a decrease in the proportion of IFN-γ positive T cells in CHB patients. IFN-γ is low in healthy serum and needs to be stimulated for the cells to secrete it in large quantities, and the results of previous experiments can only suggest an impaired function of cytokine secretion by T cells in CHB patients. Perhaps there is a decrease in the concentration of IFN-γ in inflammatory local tissue when there is a CHB infection compared to the acute infection period, but this needs further experimental.

A dendritic cell is the most common antigen presenting cell (APC). Dendritic cells (DCs) are divided into myeloid dendritic cells (myeloid dendritic cell, MDC) and serplike dendritic cells (plasmacytoid dendritic cell, PDC). DCs play important roles in the initiation and maintenance of immune cell function (37). However, the number and function of DCs change significantly in chronic hepatitis B infection (38). Our results showed that the proportion of PDC in peripheral blood of CHB patients was significantly reduced. The MDC of CD16– was not significantly different between CHB and healthy subjects, but it was different at different stages of chronic infection, suggesting that MDC showed more obvious immune deficiency during the immune tolerance period of CHB. Evidence has shown that the function of NK cells depends on their interaction with DC. On one hand, DC cells inhibit their ligand NKG2A by expressing gradually weakened HLA-E, thus activating NK cells (39). On the other hand, activated NK cells promote the development and maturation of MDC cells by secreting cytokines such as IFN-γ. Our results show that CHB patients have high expression of NKG2A and impaired NK cell IFN-γ secretion, which also supports the above argument.

Interestingly, IL-21 is the main effector factor of cTfh cells, which has a good positive correlation with the number of cTfh, and cTfh cells can achieve the regulation of B cell development through IL-21 (40). However, our study showed increased cTfh cells in CHB patients, while IL-21 secretion is decreased, suggesting that cTfh cells in CHB patients may achieve support for B cell function through other mechanisms. Studies have found that the ability of cTfh cells to produce IL-21 in response to hepatitis B surface antigen (HBsAg) during chronic hepatitis B virus infection is defective. However, cTfh cells can fully support B cells' response by producing interleukin-27 (IL-27), no matter how low IL-21 is (41). This result may provide new ideas for hepatitis B immunotherapy, and the mechanism is worth further exploration.

In summary, we found disorders in the immune cells of peripheral blood in CHB patients, especially NK cells and T cells. This phenomenon may be related to the increase of regulatory B cells and the decrease of DC cells in peripheral blood.

## Data Availability Statement

The original contributions presented in the study are included in the article/Supplementary Material, further inquiries can be directed to the corresponding author/s.

## Ethics Statement

The studies involving human participants were reviewed and approved by the Ethics Committees of the Tongde Hospital of Zhejiang Province (Identification No. 2019KY048). The patients/participants provided their written informed consent to participate in this study.

## Author Contributions

XJ and Z-hY contributed to study concept and design, acquisition of data, analysis and interpretation of data, and drafting of the manuscript. LL contributed to statistical analysis. SL contributed to samples collections. GZ and WL contributed to study concept and design, study supervision, and critical revision of the manuscript. All authors have read and approved the manuscript.

## Funding

This study was supported by the Scientific Research Fund Project of Zhejiang Traditional Chinese Medicine (Grant No.: 2017ZA024) and the Scientific Research Project of Zhejiang Province (Grant No.: 2019KY048).

## Conflict of Interest

The authors declare that the research was conducted in the absence of any commercial or financial relationships that could be construed as a potential conflict of interest.

## Publisher's Note

All claims expressed in this article are solely those of the authors and do not necessarily represent those of their affiliated organizations, or those of the publisher, the editors and the reviewers. Any product that may be evaluated in this article, or claim that may be made by its manufacturer, is not guaranteed or endorsed by the publisher.

## Abbreviations

HBV: hepatitis B virus
CHB: chronic hepatitis B
IT: immune tolerant
IC: immune clearance
LR: lower replicative
RA: reactivation
PBMC: peripheral blood mononuclear cells
APC: antigen-presenting cells
DC: dendritic cells
MFI: mean fluorescence intensity
SD: standard deviation
PDC: plasma dendritic cell
MDC: myeloid dendritic cell
cTfh: circulating T follicular helper cell
Breg: regulatory B cells.
